# Clinical Studies in the Treatment of the Nutritional Disorders of Infancy

**Published:** 1907-09

**Authors:** 


**Clinical Studies in the Treatment of the Nutritional Disorders of Infancy.** By Ralph Vincent, M.D. Pp. 83. London: Baillière, Tindall & Cox. 1906.

This little book is a supplement to the author's *Nutrition of the Infant,* published a couple of years ago, and is a series of clinical reports on infants treated in the Infants' Hospital. The object of the author is to show that slight variations in the percentages of the food stuffs make all the difference in the success or failure of infant feeding, especially in the marasmic, rachitic or otherwise abnormal babies who are brought into hospitals. The cases are rather elaborately reported, and the milk prescriptions given in full. On general principles we are in entire agreement with the author. Careful milk prescriptions either worked out on percentages of the chief constituents or on some other convenient method, are the only means we have of regulating the feeding of infants who cannot be brought up on the breast, and of restoring to health those who have been improperly dieted. The very small variations in diet percentages to which Dr. Vincent attributes so much importance do not appear to us of proved value when judged from the records in this book.

In the first place, the cases are apparently selected, not simply serial, and there is no evidence that if all the infants admitted to the Infants' Hospital were included any better results would be shown than are obtained in other Children's Hospitals, where so much stress is not laid upon minute alterations in diet. Then again, infants, even when carefully fed and looked after, show curious alterations in the rate of growth and general condition apart from small variations in diet. Dr. Vincent's own records show that the children gain and lose weight when the diet is not altered just as they do when it is. Certain comments in the book seem to us of rather doubtful advisability. For instance on p. 40, " In these cases " (presumably in half-starved infants when put on more nourishing diet) " the liver and tissues become engorged ; the liver especially comes into a condition of stasis, and the food then acts as an irritant." The exact meaning of the expression "tissues" is not clear, nor is the evidence of stasis of the liver given; apparently the fact that purgation and reduction of food effects an improvement is regarded as a proof of the correctness of the pathological theory. Again, on p. 14, describing a child who at the age of three months weighed 7 lb. 5 oz., the author remarks that the "nervous condition was due to the terrible deprivation of fat in the previous diet." Doubtless the previous diet had been very deficient in fat, but we are not aware of any evidence to show that deprivation of this food stuff alone induces any special changes in the central nervous system either organic or functional.

With Dr. Vincent's methods in general no fault can be found, and we trust that the Infants' Hospital will continue to do good work in a field where workers are much needed; but we cannot say that we are altogether convinced by the present brochure that all Dr. Vincent's views will stand the test of further experience.

